# Global SUMOylation on active chromatin is an acute heat stress response restricting transcription

**DOI:** 10.1186/s13059-015-0717-y

**Published:** 2015-07-28

**Authors:** Einari A. Niskanen, Marjo Malinen, Päivi Sutinen, Sari Toropainen, Ville Paakinaho, Anniina Vihervaara, Jenny Joutsen, Minna U. Kaikkonen, Lea Sistonen, Jorma J. Palvimo

**Affiliations:** Institute of Biomedicine, University of Eastern Finland, P.O. Box 1627, FI-70211 Kuopio, Finland; Department of Biosciences, Åbo Akademi University, Turku, Finland; Turku Centre for Biotechnology, University of Turku and Åbo Akademi University, P.O. Box 123, FI-20521 Turku, Finland; Department of Biotechnology and Molecular Medicine, A.I. Virtanen Institute, University of Eastern Finland, P.O. Box 1627, FI-70211 Kuopio, Finland; Present Address: Laboratory of Receptor Biology and Gene Expression, National Cancer Institute, NIH, Building 41, 41 Library Drive, Bethesda, MD 20892 USA

## Abstract

**Background:**

Cells have developed many ways to cope with external stress. One distinctive feature in acute proteotoxic stresses, such as heat shock (HS), is rapid post-translational modification of proteins by SUMOs (small ubiquitin-like modifier proteins; SUMOylation). While many of the SUMO targets are chromatin proteins, there is scarce information on chromatin binding of SUMOylated proteins in HS and the role of chromatin SUMOylation in the regulation of transcription.

**Results:**

We mapped HS-induced genome-wide changes in chromatin occupancy of SUMO-2/3-modified proteins in K562 and VCaP cells using ChIP-seq. Chromatin SUMOylation was further correlated with HS-induced global changes in transcription using GRO-seq and RNA polymerase II (Pol2) ChIP-seq along with ENCODE data for K562 cells. HS induced a rapid and massive rearrangement of chromatin SUMOylation pattern: SUMOylation was gained at active promoters and enhancers associated with multiple transcription factors, including heat shock factor 1. Concomitant loss of SUMOylation occurred at inactive intergenic chromatin regions that were associated with CTCF-cohesin complex and SETDB1 methyltransferase complex. In addition, HS triggered a dynamic chromatin binding of SUMO ligase PIAS1, especially onto promoters. The HS-induced SUMOylation on chromatin was most notable at promoters of transcribed genes where it positively correlated with active transcription and Pol2 promoter-proximal pausing. Furthermore, silencing of SUMOylation machinery either by depletion of UBC9 or PIAS1 enhanced expression of HS-induced genes.

**Conclusions:**

HS-triggered SUMOylation targets promoters and enhancers of actively transcribed genes where it restricts the transcriptional activity of the HS-induced genes. PIAS1-mediated promoter SUMOylation is likely to regulate Pol2-associated factors in HS.

**Electronic supplementary material:**

The online version of this article (doi:10.1186/s13059-015-0717-y) contains supplementary material, which is available to authorized users.

## Background

Heat shock (HS) induces many changes in cellular functions. Despite a general inhibition of gene expression at various levels (transcription, mRNA splicing, and translation) in response to acute heat stress or HS [1], specific genes of many cellular pathways are activated [2]. Primary responses include nuclear translocation of heat shock factors 1 and 2 (HSF1 and HSF2) and rapid activation of their target genes [3, 4].

Post-translational modifications (PTMs) of proteins allow cells to react swiftly to changing environment, which is vital for survival in acute stress situations. One of the conserved PTM responses in various types of cell stresses is the modification by small ubiquitin-like modifier (SUMO) proteins [5]. PTM of target proteins with one of three SUMO isoforms (SUMO-1/2/3 in mammals), SUMOylation, is a stepwise process in which E (enzyme) 1, the SAE1/2, activates SUMO and E2, the UBC9, conjugates the SUMO to the target protein with or without assistance from E3 ligases, such as PIAS (protein inhibitor of activated STAT) protein family members 1-4. Primary sequence of SUMO-1 is approximately 50 % identical with SUMO-2 and -3 (from now on collectively termed SUMO2/3) which are nearly identical (approximately 97 %) and indistinguishable with currently available antibodies. SUMO-2 is essential while SUMO-1 and SUMO-3 are dispensable for mouse embryonic development [6, 7]. SUMOylation has been implicated in many biological processes, especially in transcriptional regulation and DNA repair. Recent proteome-wide studies have revealed that HS induces massive SUMOylation of particularly nuclear proteins of several classes [8–10]. Many transcription factors (TFs), such as HSF1 and HSF2 [11], steroid receptors [12, 13], chromatin remodeling proteins, transcription coregulators, and subunits of RNA polymerase complexes [8–10] are known targets of HS-induced SUMOylation.

Recently, genome-wide ChIP-seq approaches have revealed cell status- and signaling-dependent alterations in the locus-selective occupancy of SUMOylated proteins and components of SUMOylation machinery [13–15]. Considering the extensive SUMOylation of chromatin binding proteins and potentially protective role of SUMOylation in HS [8], it is an open question to what extent the HS-triggered SUMOylation actually targets proteins bound to the chromatin.

To address the role of chromatin SUMOylation in HS in a genome-wide manner, we studied chromatin binding dynamics of SUMO2/3 and PIAS1 by chromatin immunoprecipitation coupled to deep sequencing (ChIP-seq) using human K562 chronic myelogenous leukemia cells and VCaP prostate cancer cells. Chromatin binding of SUMO2/3 was further correlated with HS-induced changes in on-going transcription using global run-on sequencing (GRO-seq) and chromatin occupancy of RNA polymerase II (Pol2). Our results show that HS induces rapid and massive changes in chromatin SUMOylation pattern. HS-induced SUMOylation of chromatin-bound proteins correlates positively with transcriptional activity at promoters and enhancers. Promoter SUMOylation is dependent on active transcription, as inhibition of transcription initiation or elongation reduces the amount of SUMO2/3 at promoters. Mechanistically, SUMOylation seems to prevent hyperactivation of HS-induced transcription, as silencing of the SUMOylation machinery leads to significantly increased gene expression of HSPs in cells exposed to acute heat stress.

## Results

### Heat shock induces changes in chromatin SUMOylation pattern

**Fig. 1:**
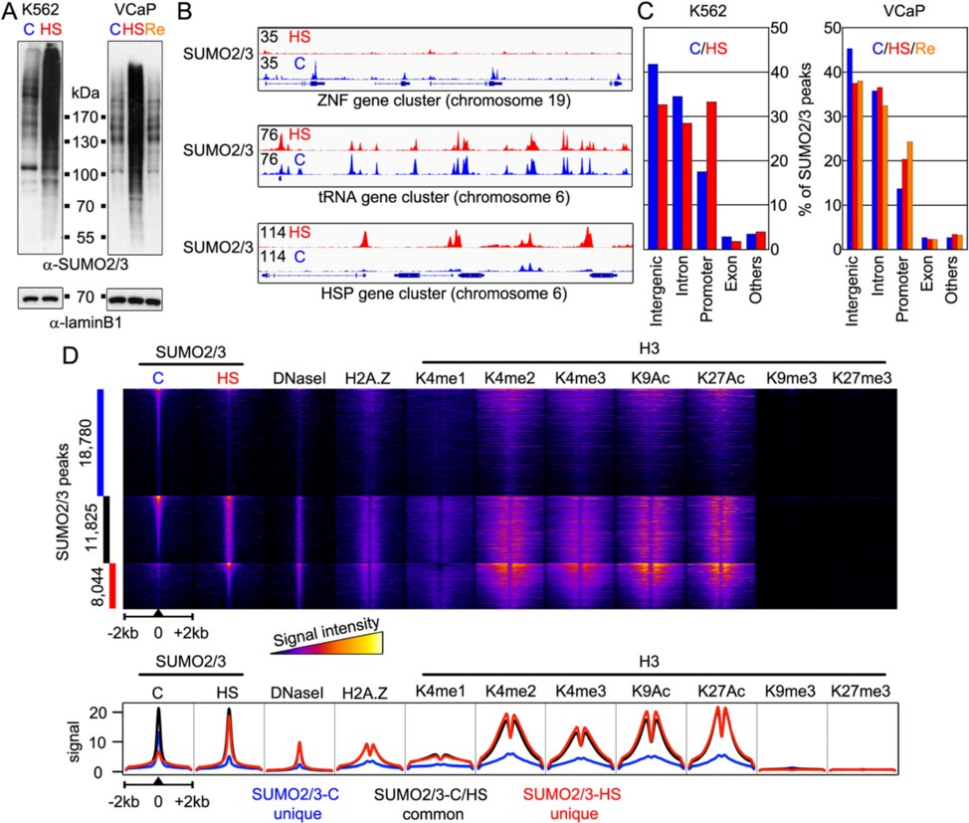
Redistribution of chromatin-bound SUMO2/3 in heat stress. **a** SUMO2/3-modified cellular proteins accumulate in K562 and VCaP cells upon heat stress. Anti-SUMO2/3 antibody immunoblotting analysis of total cellular lysates from cells grown at control conditions (C; 37 °C) or exposed to heat shock (HS; 30 min at 43 °C), and from VCaP cells in recovery from HS (Re; HS followed by 1 h at 37 °C). Anti-lamin B1 antibody was used as a loading control. **b** Redistribution of chromatin SUMO2/3 in HS. SUMO2/3 (anti SUMO-2/3 antibody) ChIP-seq track examples of chromatin loci in K562 cells where SUMO2/3-binding decreases (ZNF gene cluster), is unaltered (tRNA gene cluster), or increases (HSP (heat shock protein) gene cluster) in HS. Numbers (on the left) depict maximal signal intensities of each track. **c** Distribution of SUMO2/3 peaks between annotated genomic loci in K562 and VCaP cells. SUMO2/3 peaks are from in control (blue), HS (red), and recovery (orange) conditions. **d** Chromatin environment of SUMO2/3 peaks categorized to SUMO2/3 C unique peaks (blue bar), SUMO2/3 C/HS-shared peaks (black bar), and SUMO2/3 HS unique peaks (red bar). Numbers on the left refer to the amount of peaks in each category. Heat map showing SUMO2/3-binding site, DNaseI and different histone mark signals in ±2 kb window using false-color scale (intensity increases from darker to lighter colors). Line profile of average ChIP-seq signal intensities at ±2 kb area surrounding the peak centers of different SUMO2/3 categories. ChIP-seq signals were normalized to 10 million reads

To investigate HS-induced changes in chromatin SUMOylation, we exposed K562 cells to 43 °C for 30 min. The exposure induced a prominent accumulation of high molecular mass anti-SUMO2/3 immunoreactive protein forms, as judged by immunoblot analysis of whole cell lysates (Fig. 1a). The HS in VCaP cells (30 min at 43 °C) caused a similar increase in total cellular SUMOylation that was reversed after 60 min recovery at 37 °C (Fig. 1a). To monitor how HS-induced changes in SUMOylation are reflected on chromatin, we performed ChIP-seq analyses using the same anti-SUMO-2/3 antibody in K562 and VCaP cells kept at control temperature (37 °C, SUMO2/3-C) or exposed to HS (SUMO2/3-HS). In addition, ChIP-seq was used to monitor chromatin SUMOylation changes in VCaP cells during recovery from HS (SUMO2/3-Re). Alignment of ChIP-seq signal against human hg19 genome revealed strong SUMO2/3 signals, representing SUMO2/3-enriched sites (binding sites or peaks), in both control and HS conditions (Fig. 1b). Interestingly, HS induced striking changes in the intensity of SUMO2/3 signal in many, but not all, chromatin loci: some sites lost (for example, zinc finger gene cluster), while others retained (for example, tRNA gene cluster) or accumulated (for example, HSP gene cluster) SUMO2/3 upon HS (Fig. 1b).

Analysis of SUMO2/3 peaks in K562 cells that were found in two independent biological replicates yielded in control conditions 30,605 SUMO2/3 peaks and in HS 19,869 SUMO2/3 peaks of which approximately 12,000 occurred in both conditions. In VCaP cells, the ChIP-seq analysis revealed 10,154 SUMO2/3 peaks in control cells, 25,382 in heat-shocked cells, and 19,132 in HS-recovered cells. In both cell lines, the majority of the SUMO2/3 peaks were found in intergenic, intron, and promoter regions in both conditions (Fig. 1c). The most notable change in HS was the increase in promoter-associated SUMO2/3 peaks: from 17.5 % to 33.2 % in K562 cells and 13.7 % to 20.3 % in VCaP cells. In VCaP cells, the proportion of promoter-associated SUMO2/3 peaks was further increased during the recovery from heat stress (24.2 %; Fig. 1c). Comparison of K562 and VCaP SUMO2/3 peaks showed a modest overlap in control condition (approximately 2,900 peaks) that increased in HS (approximately 4,700) (Additional file 1: Fig. S1). Of note, >70 % of the HS SUMO2/3 peaks co-occurring in both cell lines were found at promoters.

We utilized the available ENCODE data of K562 cells [16] and analyzed markers of active and repressed chromatin in the vicinity of SUMO2/3 peaks. The K562 SUMO2/3 peaks were clustered to three categories (Fig. 1d): control unique peaks (blue bar), peaks shared with control and HS (black bar), and HS unique peaks (red bar). SUMO2/3 HS unique peaks showed signs of open chromatin (DNaseI accessibility) and strong signals from histone marks H2A.Z, H3K4me1/2/3, H3K9Ac, and H3K27Ac that are associated with active chromatin [17]. The same chromatin signature was found in the vicinity of SUMO2/3 peaks shared with control and HS conditions, but to a much lesser extent in control unique SUMO2/3 peaks. All SUMO2/3 peaks were devoid of the histone marks H3K9me3 and H3K27me3 that are associated with repressed chromatin [17].

Taken together, the majority of the SUMO2/3 peaks is cell type-specific and located in introns and intergenic regions. Acute heat stress changed chromatin SUMOylation pattern rapidly: SUMO2/3 accumulated onto promoters and chromatin regions with active histone marks and it was lost from intergenic chromatin sites.

### Characterization of SUMO2/3-binding chromatin sites

**Fig. 2:**
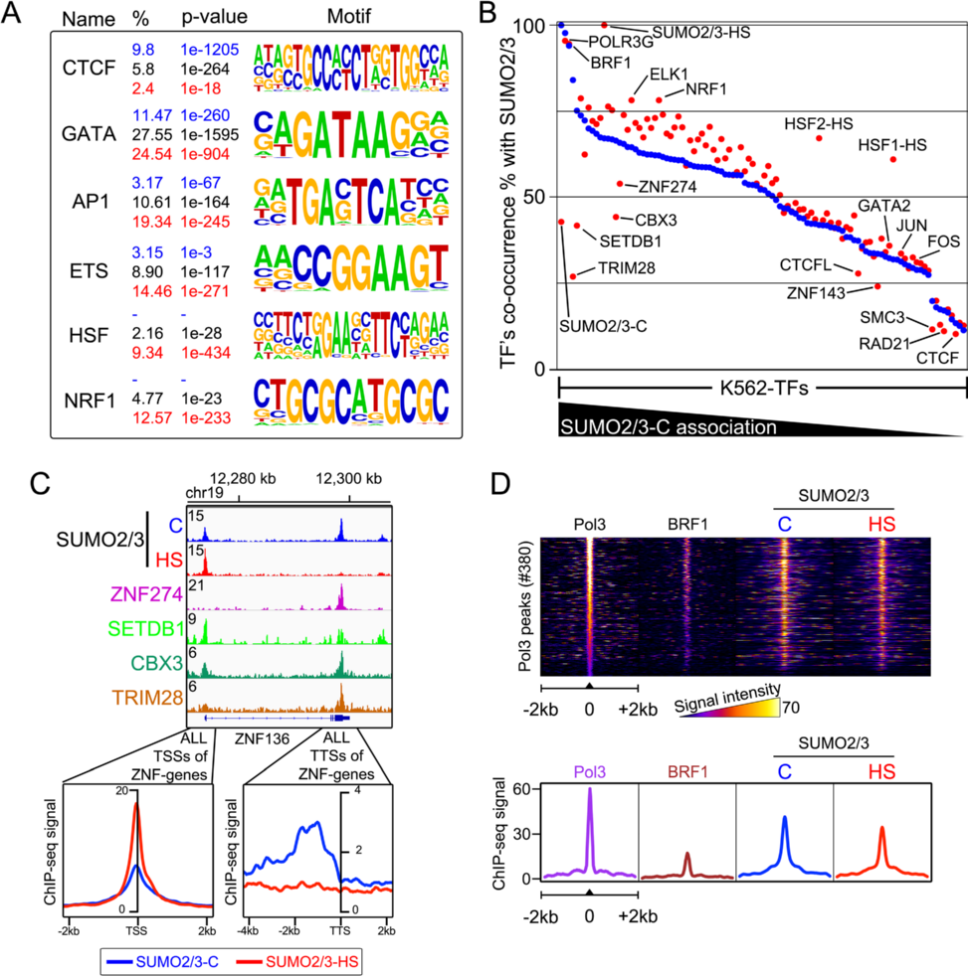
Characterization of SUMO2/3 chromatin binding sites in K562 cells. **a** Enrichment of DNA-binding motifs within SUMO2/3 peaks. Percentage of SUMO2/3 peaks with the motif and *P* value of enrichment are indicated with color code (C unique, blue; C/HS-shared, black; SUMO2/3 HS, red). **b** Co-occurrence of K562 TFs and SUMO2/3 peaks. Scatter plot of TF co-occurrence with SUMO2/3 C (blue dots) and SUMO2/3 HS (red dots) peaks. Y-axis represents co-occurrence percent of analyzed TF and on X-axis all analyzed TFs are arranged according to their co-occurrence percent with SUMO2/3 C. Complete list of analyzed K562-TFs and co-occurrence percentages in Table S1. **c** SUMO2/3 occupancy at ZNF genes. SUMO2/3 ChIP-seq track in control (C) and HS conditions aligned with ChIP-seq tracks of ZNF274, SETDB1, CMX3, and TRIM28 at *ZNF136* locus. Comparison of SUMO2/3 signal at TSSs and TTSs of all annotated ZNF genes in C and HS conditions. **d** Association of SUMO2/3 and Pol3. Heat map of ±2 kb window centered at Pol3 binding sites and showing ChIP-seq signals of Pol3 (POLR3G subunit), Pol3-associated TF BRF1, and SUMO2/3 in C and HS conditions using false-color scale. Line profile of average ChIP-seq signals in ±2 kb window from the centers of Pol3-binding sites

Based on heat map and line profile analyses at the peak vicinities (Fig. 1d), the SUMO2/3 signal was confined to small chromatin regions, suggesting that SUMO2/3 signal derives from occupancy of SUMOylated transcription factors on chromatin. To gain insight into potential SUMO2/3 acceptor proteins on the chromatin, we analyzed the DNA sequences at SUMO2/3 peaks from K562 cells for the enrichment of conserved binding motifs. CCCTC-binding factor (CTCF) and GATA motifs were ubiquitous in control unique SUMO2/3 peaks and in peaks shared with control and HS (Fig. 2a; full list of associated motifs in Additional file 2: Table S1). Activator protein 1 (AP1) and E-twenty-six (ETS) motifs were more prevalent in HS compared to control SUMO2/3 peaks. Of note, HSF1 and nuclear respiratory factor 1 (NRF1) motifs were highly enriched only in HS unique SUMO2/3 peaks (Fig. 2a).

Next, we compared the co-occurrence of SUMO2/3 peaks with K562 cell chromatin binding protein data from the ENCODE project [16, 18] and with HSF1 and HSF2 HS ChIP-seq data [19]. Co-occurrence was analyzed by calculating the percentage of peaks in each chromatin-binding dataset (collectively termed K562 TFs) that is overlapping with SUMO2/3 peaks. Strikingly, >50 % co-occurrence with SUMO2/3 was found for nearly half (45/103) of K562 TFs (Fig. 2b; full list of co-occurrences in Additional file 3: Table S2). Average co-occurrence frequency of the K562 TFs with SUMO2/3 peaks was similar in control and HS cells (control: 48.9 %; HS: 51.2 %), which is remarkable, as there were 35 % less SUMO2/3 peaks in HS cells. All analyzed ENCODE datasets are from normal (37 °C) growth conditions, while the HSF1 and HSF2 data are from K562 cells exposed to acute heat stress. In agreement with the motif analysis, the most notable HS-induced increase in SUMO2/3 co-occurrence was seen with HSF1 and HSF2. Also NRF1 and ELK1 co-occurred with SUMO2/3 more frequently in HS, and a minor increase in the co-occurrence was observed with many proteins, including the AP1 subunits JUN and FOS. Conversely, HS markedly reduced the co-occurrence of SUMO2/3 with several proteins that are components or associate with CTCF-cohesin complex (CTCF, RAD21, SMC3, and ZNF143) [20, 21], and histone H3 lysine-9 (H3K9) methylation machinery (ZNF273, SETDB1, and TRIM28) [22]. CTCF, cohesin, and ZNF143 are often, but not exclusively, binding the same loci on chromatin, and these co-bound loci are suggested to control chromatin 3D structure [23]. Almost one-third (29.9 %) of SUMO2/3 sites that were lost in HS are occupied by at least one of these factors and many (13.0 %) by all three (Additional file 1: Fig. S2). Targets of the H3K9 methylation machinery include the 3’ ends of zinc finger (ZNF) genes at which SUMOylation of the transcription factor TRIM28 has been reported to be required for positioning of SETDB1 [24]. In line with previous observations [14], we observed a prominent SUMO2/3 signal at the 3’ ends close to the transcription termination sites (TTSs) of ZNF genes, and comparison with the ENCODE data showed that this SUMO2/3 signal overlaps with the signals of TRIM28, CBX3, SETDB1, and ZNF247 in control conditions (for example, *ZNF136* gene, Fig. 2c). Interestingly, at the TTSs, SUMO2/3 signal disappeared in HS, and the analysis of all 538 annotated ZNF genes revealed that this was a general phenomenon (Fig. 2c; histogram). The loss of SUMO2/3 signal at ZNF genes was specific for the 3’ ends, as the signal at the transcription start sites (TSSs) contrastingly increased in HS compared to control cells (Fig. 2c).

Some transcription factors in K562 cells showed a very high co-occurrence with SUMO2/3 peaks, suggesting that their regulation or chromatin binding is tightly linked to SUMOylation. The highest co-occurrence with SUMO2/3 was observed with RNA polymerase III (Pol3) and its associated transcription factor BRF1, and the situation prevailed in HS (control, 97.7 %; HS, 95.5 %; Fig. 2b). Heat map and line profile analysis of all 380 Pol3-binding sites revealed that chromatin SUMO2/3 signal peaked at the center of the binding sites (Fig. 2d). The overall SUMO2/3 signal at Pol3-binding sites decreased slightly in HS (Fig. 2d).

Taken together, our bioinformatic analyses link HS-induced chromatin SUMOylation changes to several proteins that are thus likely to change their SUMOylation status or chromatin binding upon HS. SUMOylation was often lost from sites that bind the CTCF-cohesin-complex or H3K9 methylation machinery, including 3’-binding sites at ZNF genes. Heat stress increased the SUMO2/3 co-occurrence of the majority of analyzed transcription factors, suggesting that SUMO-mediated regulation at chromatin regulatory regions is a common HS response. The most prominent increase in SUMO2/3 co-occurrence in HS was detected for HSF1 and HSF2.

### Role of HSF1 in HS-induced changes in chromatin SUMO2/3 pattern

**Fig. 3:**
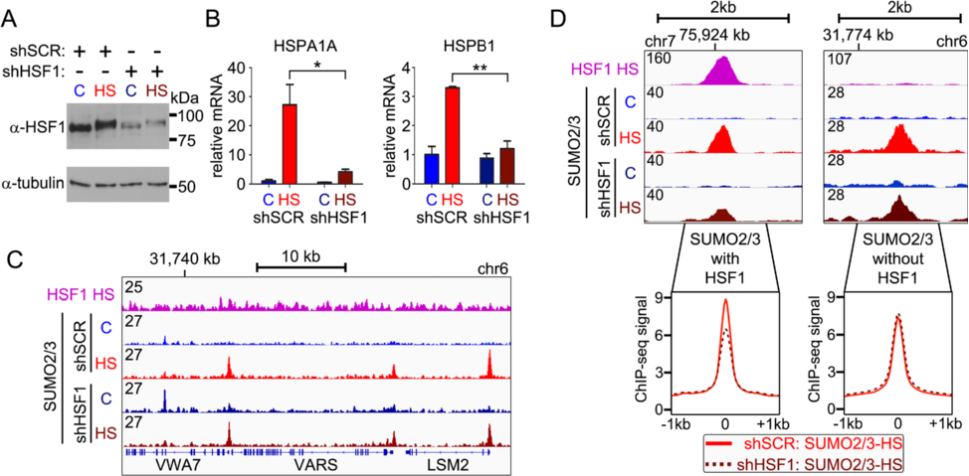
Effect of HSF1 depletion on chromatin SUMO2/3 pattern in K562 cells. **a** Anti-HSF1 antibody immunoblotting of total cellular lysates from shSCR (scrambled control)- or shHSF1-treated cells in C or HS conditions. Anti-tubulin was used as a loading control. **b** HS-triggered induction of HSP gene expression is blunted in HSF1-silenced (shHSF1) cells. RT-qPCR with primers specific for *HSPA1A* and *HSPB1* was used to measure gene expression. Error bars represent standard deviation of three biological replicates. Stars depict statistical significances between indicated samples (Student’s *t*-test, **P <0.01, *P <0.05). **c** HS-induced changes in chromatin SUMO2/3 are generally independent on HSF1 silencing. ChIP-seq tracks of HSF1 in HS, and SUMO2/3 in C and HS conditions in shSCR- and shHSF1-treated cells at indicated chromatin loci. Numbers (on the left) depict maximal signal intensity of each track. **d** HSF1/SUMO2/3 co-bound loci lose SUMO2/3 signal in HSF1 depleted cells. Two intergenic HS-inducible SUMO2/3 loci with and without HSF1-binding site. Comparison of average SUMO2/3 HS ChIP-seq signals in ±1 kb window at the centers of SUMO2/3 and HSF1 co-occupied peaks and SUMO2/3 peaks without HSF1 in shSCR- or shHSF1-treated cells as indicated

To study the role of HSF1 in the HS-triggered SUMO2/3 chromatin redistribution, we silenced HSF1 in K562 cells and analyzed the effect of silencing on chromatin SUMOylation. Transfection of a short-hairpin RNA against HSF1 (shHSF1) markedly reduced HSF1 protein level (Fig. 3a) and inhibited the activation of HSP genes upon HS (Fig. 3b), when compared to non-silenced (shSCR-transfected) and heat-shocked cells. SUMO2/3 ChIP-seq showed that the overall HS-induced changes in chromatin SUMO2/3 signal were largely unchanged in HSF1-silenced cells, indicating that HSF1-regulated processes are not needed for the general HS-induced chromatin SUMO2/3 response (Fig. 3c). However, HSF1 binding is rapidly induced in HS [19], and >60 % of the HSF1 peaks co-occurred with SUMO2/3 in HS (Fig. 2b), indicating that these chromatin SUMO2/3 signals could derive from SUMOylated HSF1. Indeed, in HS, the intensity of SUMO2/3 signal at the SUMO2/3 peaks that co-occurred with HSF1 was reduced in HSF1-silenced cells (Fig. 3d; SUMO2/3 with HSF1), whereas no reduction was seen at SUMO2/3 peaks that did not co-occur with HSF1 (Fig. 3d; SUMO2/3 without HSF1). These data support the notion that the majority (approximately 95 %) of the changes in chromatin SUMOylation represent direct signaling responses to heat stress and that they are not dependent on the activation of HSF1 or its target genes.

### Chromatin SUMOylation in HS is associated with Pol2 pausing and global reprogramming of transcription

**Fig. 4:**
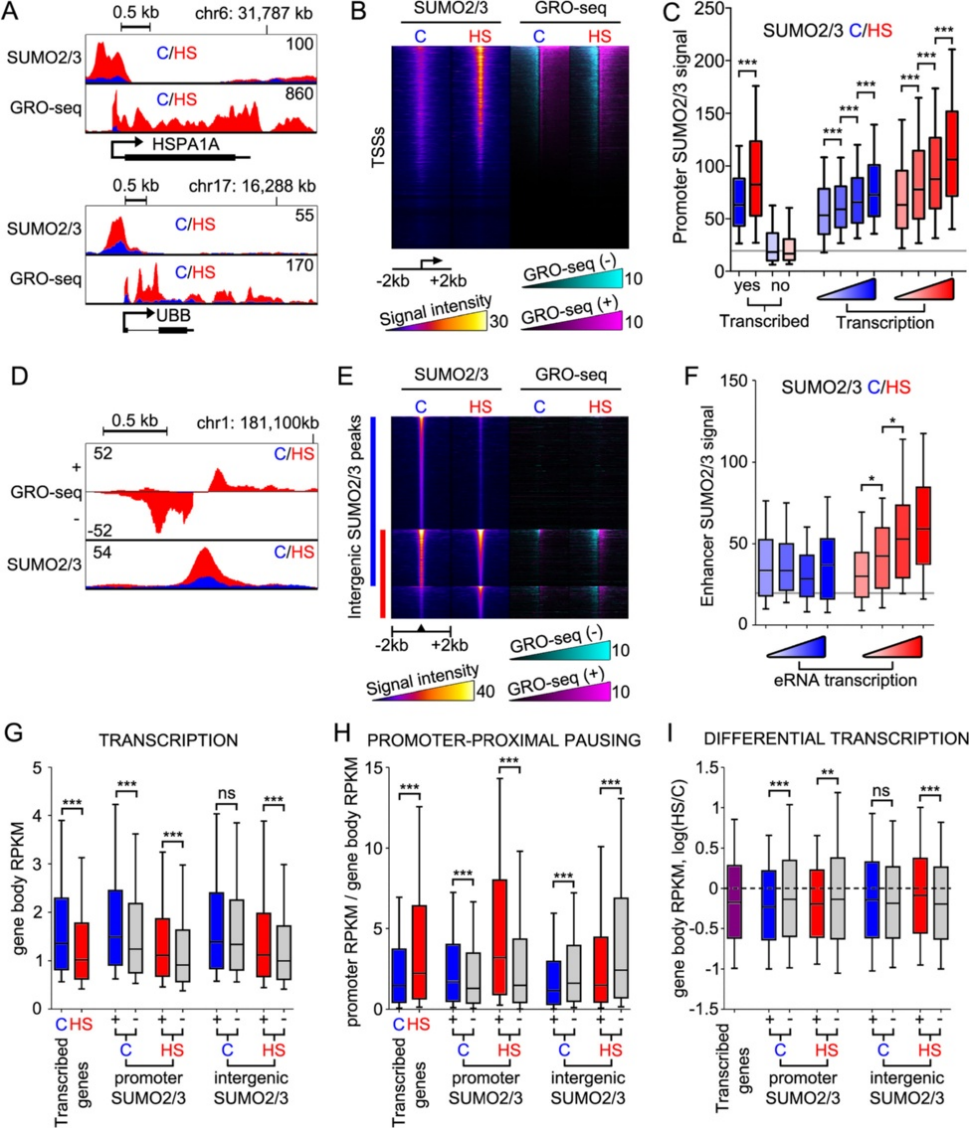
Transcription-tied-up SUMOylation in HS. **a** GRO-seq (plus-strand) and SUMO2/3 ChIP-seq signals at HS-inducible *HSPA1A* and *UBB* loci in K562 cells in control (blue) and in HS conditions (red). Numbers depict the maximum signal. **b** Heat map showing SUMO2/3 ChIP-seq (in ±2 kb window using false-color scale) and GRO-seq signal densities (minus-strand, cyan; plus-strand, magenta) at TSSs arranged according to control GRO-seq signal. **c** Whisker-plot of SUMO2/3 control (blue boxes) and HS (red boxes) ChIP-seq signals at the promoters of transcribed and non-transcribed genes, and at promoters of genes divided into four groups of 2,417 genes according to increasing transcription in control or in HS. Gray line indicates background signal intensity. **d** GRO-seq plus- and minus-strand signals and SUMO2/3 signals in control (blue) and HS (red) at HS-inducible intergenic SUMO2/3 peak. **e** Heat map showing the same as in (b) but (instead for TSSs) for intergenic SUMO2/3-binding sites. Heat map is divided into three parts according to control (C; blue bar) and HS (red bar) SUMO2/3-binding sites. **f** Whisker-plot of SUMO2/3 control (blue boxes) and HS (red boxes) ChIP-seq signals at four groups of 135 eRNA enhancers with increasing transcription in control or HS. Gray line indicates background signal intensity. **g** Correlation between gene transcription and SUMO2/3 binding. Whisker-plot showing gene body GRO-seq signal (in RPKM (reads per kilobase per million mapped reads)) of transcribed genes in C and HS conditions. In each comparison, transcribed genes were divided into two groups based on their promoter or intergenic SUMO2/3 association (+, association; -, no association) in control (blue) or HS conditions (red). Gray depicts transcribed genes not associated with particular SUMO2/3 category. **h** Correlation between promoter pausing and SUMO2/3 binding. Promoter pausing index (PPI) was calculated by dividing promoter GRO-seq RPKM value with gene body RPKM value. SUMO2/3 peak associations are the same as in (g). **i** Correlation between SUMO2/3 binding and HS-induced changes in transcription. SUMO2/3 peak associations are the same as in (g). HS-induced change in gene transcription was measured from gene-body GRO-seq signal (gene body RPKM in HS divided by gene body RPKM in control) and shown as log_2_(HS/C). In all whisker-blots, stars depict *P* values (***P <0.001, **P <0.01, ns = not significant) of Student’s *t*-test (g, h, i) or Kruskall-Wallis/Dunn’s multiple comparison tests (c, f). Line represents median, box edges are 25 % and 75 % boundaries, and whiskers 10 % and 90 % of the data

SUMO2/3 peaks were prominently associated with active chromatin marks (Fig. 1d), and hence, we analyzed the correlation between chromatin SUMO2/3 and transcription in a genome-wide fashion. We mapped the ongoing transcription from control and HS-exposed K562 cells using global run-on sequencing (GRO-seq) [25]. The HS-induced activation of HSP genes was accompanied by an increase in the promoter SUMO2/3 signal, initially suggesting that promoter SUMO2/3 signal stimulates gene activation (for example, *HSPA1A* and *UBB* in Fig. 4a). Analysis of all TSSs, however, showed that promoter SUMO2/3 signal was broadly present and substantially increased in HS at the promoters of transcribed genes, implying that enhanced promoter SUMOylation is a global response to acute heat stress and not restricted to HS-activated genes (Fig. 4b). Indeed, SUMOylation was markedly increased in HS at promoters of transcribed genes (gene body RPKM >0.5 in control or HS GRO-seq), whereas SUMOylation remained at the background level at the promoters of non-transcribed genes (Fig. 4c). When we divided the actively transcribed genes into four groups of increasing transcription, a positive correlation between promoter SUMOylation and transcription was apparent in control (r_s_ (spearman correlation coefficient) = 0.21, *P* value <0.0001) and HS (r_s_ = 0.27, *P* value <0.0001; Fig. 4c). Furthermore, the SUMO2/3 signal overlapped with Pol2 occupancy on the promoters, albeit the SUMO2/3 peaked somewhat upstream from the major Pol2-binding site (Additional file 1: Fig. S3).

Active enhancers have been shown to produce short bidirectional RNAs, eRNAs, and the amount of these RNAs is known to correlate with the enhancer activity [26, 27]. The intergenic SUMO2/3 peaks were frequently associated with HS-induced short bidirectional transcription (Fig. 4d). Analysis of all intergenic SUMO2/3 peaks showed that bidirectional transcription was often centered at the HS SUMO2/3 peaks, whereas the SUMO2/3 peaks unique to control conditions did not show such transcription (Fig. 4e). In addition, intergenic SUMO2/3 peaks in HS, but not those unique to control condition, were enriched with histone marks of active chromatin (Additional file 1: Fig. S4). To further analyze the connection between enhancer activity and SUMO2/3, we defined intergenic chromatin loci that produce bidirectional transcripts as eRNA enhancers. These 539 loci were further divided into four groups according to increasing transcription level and SUMO2/3 signal within each group was analyzed (±500 bp region from the center of eRNA enhancer; Fig. 4f). The analysis showed that the amount of eRNA transcription is paralleled by enhancer SUMOylation in HS (r_s_ = 0.36, *P* <0.0001), but not in control conditions (Fig. 4f). These data suggest that upon exposure to acute heat stress, the intergenic SUMO2/3 peaks correspond to active or activated enhancers.

We next asked if genes that have promoter or adjacent intergenic SUMO2/3 peaks respond to HS differently than genes without SUMO2/3 peaks. In addition to analyzing HS-induced changes in transcription, we measured Pol2 promoter-proximal pausing, which is one of the key mechanisms regulating transcription in HS [28, 29]. Pol2 pausing is measured by using promoter-proximal pausing index (PPI), which is the ratio of promoter-proximal transcription and gene-body transcription. We classified transcription to promoter proximal (from TSS to +250 bp) and gene body regions to measure the PPI and transcription (Additional file 1: Fig. S5). As an early response to HS, 1,730 genes were found to be differentially transcribed (795 induced and 935 repressed; false discovery rate, FDR <0.01; fold change >2). Thus, HS typically resulted in repression of transcription (Fig. 4g, i) and an increase in PPI (Fig. 4h) at transcribed genes. Nearly half of the transcribed genes had a SUMO2/3 peak at their promoters (44.0 % in control, 51.1 % in HS) and approximately one-fifth were associated (TSS within 100 kb) with an intergenic SUMO2/3 peak (21.0 % in control, 14.3 % in HS; Additional file 1: Fig. S6). In control conditions, promoter SUMO2/3-associated genes were more actively transcribed (promoter SUMO2/3-C [+] in Fig. 4g) and displayed higher PPIs (Fig. 4h) than genes without promoter SUMO2/3 association (promoter SUMO2/3-C [-]). Promoter SUMO2/3 peak-associated genes had significantly higher PPIs compared to genes without a promoter SUMO2/3 peak, especially in HS (Fig. 4h). In addition, transcription of genes with promoter-associated SUMO2/3 in either control or HS was more severely repressed in HS than genes without the promoter SUMO2/3 (Fig. 4i). Contrary to promoter SUMO2/3 association, intergenic SUMO2/3-associated genes showed lower PPIs both in control and HS conditions when compared to unassociated genes (Fig. 4h). In HS, intergenic SUMO2/3-associated genes were also more actively transcribed (Fig. 4g) and less repressed (Fig. 4i).

We also identified the biological processes affected in HS and those associated with HS-induced changes in chromatin SUMOylation. The HS-induced genes were enriched with Gene Ontology (GO) biological process terms related to protein folding, apoptosis, and heat response, while the HS-repressed genes were enriched in GO terms of gene expression, transcription, translation, and metabolic processes (Table 1 for representative GO terms and full list in Additional file 4: Table S3). HS-enriched SUMO2/3 peaks (2,739 peaks with >4-fold signal in HS than in control) were also associated with GO terms of translation and RNA processes (Table 1).

**Table 1:** Enrichment of gene ontology (GO) terms among HS-induced and HS-repressed genes and that of GO terms among HS-enriched and HS-depleted SUMO2/3 peaks in K562 cells

Gene group	GO term	Genes (n)	*P* value	B-H FDR
HS-induced	Response to unfolded protein	21	1.7E-14	4.2E-11
	Protein folding	23	2.3E-08	1.1E-05
	Programmed cell death	41	4.6E-06	1.2E-03
	Apoptosis	39	1.8E-05	4.4E-03
	Response to heat	10	4.5E-05	9.9E-03
HS-repressed	Gene expression	225	1.3E-17	3.2E-14
	Metabolic process	442	1.7E-16	2.8E-13
	Biosynthetic process	251	1.9E-16	1.4E-13
	Transcription	155	8.7E-11	1.6E-08
	Translation	35	5.4E-06	5.1E-04
	Translational elongation	16	4.0E-05	3.6E-03
	ncRNA metabolic process	24	2.6E-04	2.1E-02
SUMO2/3 peak group	GO term	Peaks (n)	*P* value	Bonferroni *P* value
HS-enriched	Translation	183	1.1E-38	1.0E-34
	mRNA metabolic process	278	6.7E-36	5.9E-32
	RNA processing	273	3.5E-31	3.1E-27
	Viral reproduction	175	1.2E-29	1.1E-25
	Translational elongation	80	2.6E-29	2.3E-25
	Viral transcription	63	1.5E-27	1.3E-23
	Translational termination	64	8.7E-27	7.6E-23
	ncRNA metabolic process	132	1.3E-23	1.1E-19
	Protein complex disassembly	70	1.3E-22	1.1E-18
	RNA splicing	136	3.3E-14	2.9E-10
HS-depleted	Regulation of lipid kinase activity	27	2.9E-06	2.1E-02

Taken together, HS triggered transcription-tied-up SUMOylation of promoters and enhancers. Promoter SUMOylation correlated positively with Pol2 promoter-proximal pausing and predicted transcriptional repression upon acute heat stress. Moreover, genes associated with the HS-enriched SUMO2/3 peaks shared several of the biological process categories of the HS-repressed genes.

### HS-induced dynamic binding of SUMO ligase PIAS1 onto Pol2-bound promoters

**Fig. 5:**
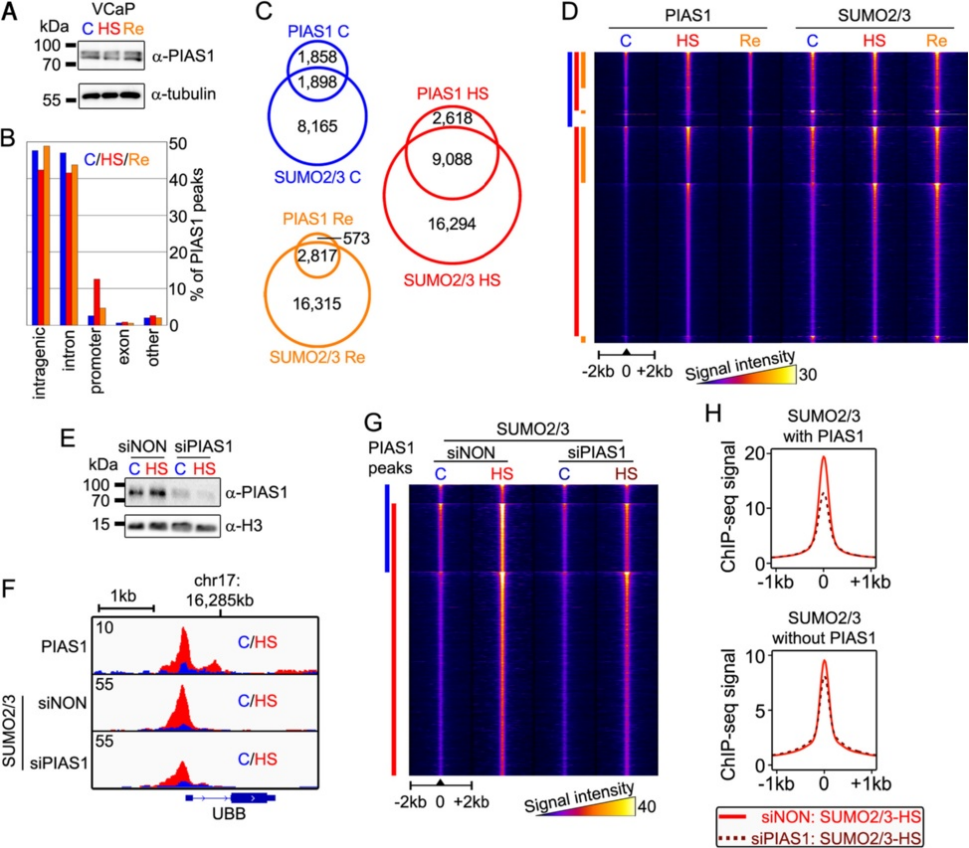
Chromatin-bound PIAS1 promotes SUMOylation on VCaP cell chromatin. **a** Total cellular PIAS1 levels do not change in 30 min HS at 43 °C (HS) or 1 h recovery at 37 °C (Re), as judged by immunoblotting of total cellular lysates with anti-PIAS1 antibody. Anti-tubulin antibody was used as a loading control. **b** Genomic location of SUMO2/3 peaks in each condition in annotated chromatin loci. **c** Venn-diagrams showing overlap of PIAS and SUMO2/3 peaks in control (C), HS (HS), and recovery (Re) conditions. **d** Heat map showing PIAS1 and SUMO2/3 ChIP-seq signal intensities in control (C), HS, and recovery (Re), using false-color scale in ±2 kb windows centered at PIAS1 binding sites. Blue bar depicts PIAS1 peak cluster in control conditions, red bars clusters in HS, and orange bars in recovery. **e** Depletion of PIAS1 with specific siRNA (siPIAS1) as confirmed by immunoblotting (siNON, non-targeting control siRNA). Anti-histone 3 (H3) was used as a loading control. **f** SUMO2/3 ChIP-seq signal was reduced at PIAS1 co-occurring chromatin sites, such as the promoter of HS-inducible *UBB*, in PIAS1-depleted cells. ChIP-seq signals in C and HS conditions are marked with blue and red, respectively. **g** Heat map showing SUMO2/3 ChIP-seq signal in siNON- and siPIAS1-transfected cells in C and HS conditions at ±2 kb window centered at PIAS1 binding. ChIP-seq signal intensities are shown as false-color. Bars represent PIAS1-binding sites in C (blue) and HS (red) conditions. **h** Comparison of average HS SUMO2/3 ChIP-seq signals at SUMO2/3 peaks with or without co-occurring PIAS1 in siNON- (red line) and siPIAS1- (dark red dotted line) transfected cells

PIAS1 is the major PIAS family member in VCaP cells and known to bind chromatin [15]. To get insight into the chromatin binding dynamics of the SUMOylation machinery in HS, we mapped genome-wide chromatin binding of PIAS1 in control, HS (30 min at 43 °C) and recovery (HS followed by 60 min at 37 °C) in VCaP cells using ChIP-seq. Overall PIAS1 protein levels did not change upon HS or recovery (Fig. 5a). The number of PIAS1 peaks, however, increased over three-fold in HS (from 3,756 to 11,706 peaks) and returned to the control level after recovery period (3,390 peaks). Although PIAS1 peaks were predominantly found at intergenic regions or introns (Fig. 5b), the most dramatic HS-induced change was detected at the promoter regions where the number of PIAS1 peaks increased by 15-fold (Fig. 5b; 98 peaks vs. 1,470 peaks). The number of PIAS1 peaks that co-occurred with SUMO2/3 increased almost five-fold from control (1,898 peaks) to HS (9,088 peaks) (Fig. 5c). Moreover, overall intensity of PIAS1 signal in HS was stronger than that in recovery (or control) at the binding sites shared between HS, control and recovery conditions, underlining the dynamic nature of HS-induced chromatin binding of PIAS1 (Fig. 5d). SUMO2/3 signal at the HS-induced PIAS1-binding sites increased in HS and remained high after dissociation of PIAS1 in recovery.

The above data suggest that HS induces activation and chromatin binding of PIAS1 where it functions as a SUMO ligase. To test this notion, we silenced PIAS1 using siRNA (siPIAS1) (Fig. 5e) and analyzed how this affects occupancy of SUMO2/3 on chromatin. The silencing resulted in a reduction of SUMO2/3 at many PIAS1 co-occurring loci in HS (Fig. 5f, g; SUMO2/3 siNON vs. siPIAS1). On average, SUMO2/3 signal at PIAS1 co-occurring sites (Fig. 5h; SUMO2/3 with PIAS1) was reduced by approximately 33 %, while only <15 % reduction was seen at SUMO2/3 sites that did not co-occur with PIAS1 (SUMO2/3 without PIAS1).

### Correlation of transcription with promoter SUMOylation and PIAS1 binding

**Fig. 6:**
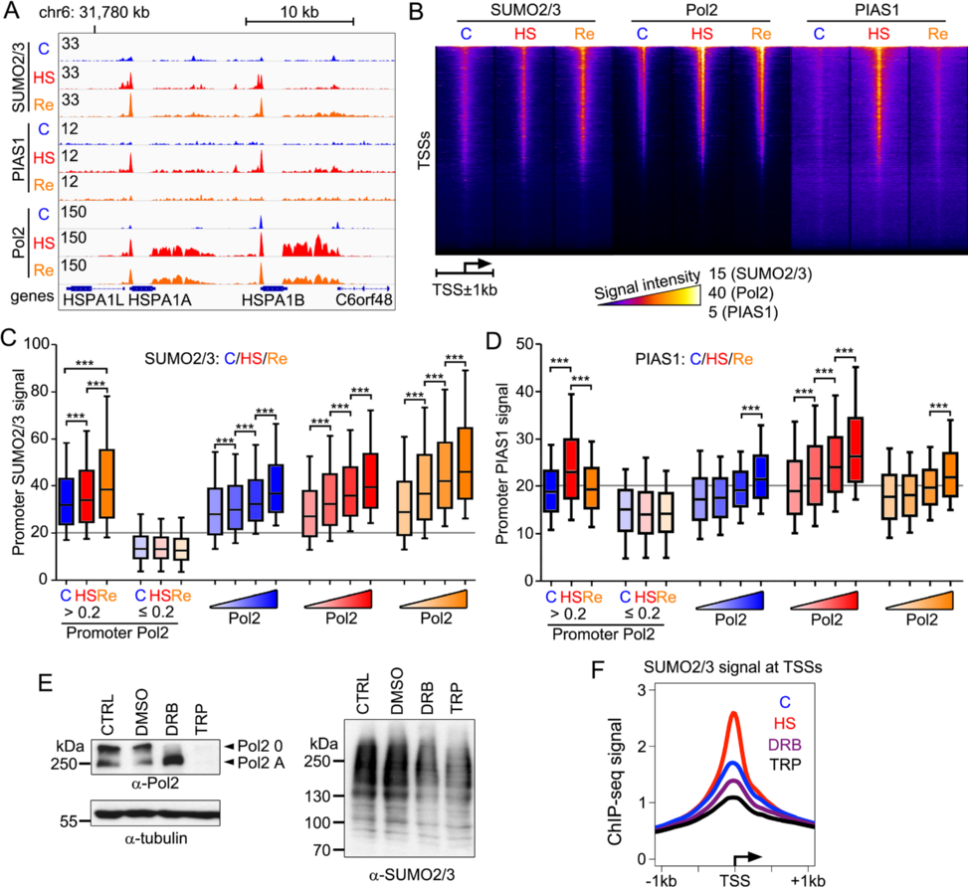
Positive correlation between promoter-bound Pol2, SUMO2/3 and PIAS1. **a** SUMO2/3, PIAS1, and Pol2 ChIP-seq tracks at HSP locus in VCaP cells in control (blue), HS (red), and recovery (orange) conditions. **b** Heat map showing SUMO2/3, PIAS1, and Pol2 ChIP-seq signals in control (C), HS, and recovery (Re) at TSS ± 1 kb window centered to annotated TSSs and arranged according to Pol2 signal in HS. Intensities are presented using false-color scale. Maximum signals for SUMO2/3, PIAS1, and Pol2 are 15, 5, and 40, respectively. **c** Promoter SUMO2/3 occupancy positively correlates with promoter Pol2 occupancy. Genes were classified to active (RPKM >0.2) and silent (RPKM ≤0.2) based on their promoter Pol2 signal. Promoter SUMO2/3 signal on active genes increased significantly from C via HS to Re. SUMO2/3 signal was below background level (gray line, median IgG ChIP-seq signal at promoters) at promoters of silent genes. Promoter SUMO2/3 signal increased with increasing promoter Pol2 signal when genes were divided into four categories of 4,051 genes based on their promoter Pol2 signal in control (blue), HS (red), or recovery (orange) conditions. **d** Promoter PIAS1 occupancy correlates with that of Pol2 only in HS. Gene categories are the same as in (c). **e** Immunoblotting with anti-Pol2 antibody shows that the presence of both hyperphosphorylated (Pol2 0) and non-phosphorylated (Pol2 A) in non-treated (CTRL) and vehicle-treated (DMSO) cells. DRB (100 μM, 3 h) efficiently inhibits hyperphosphorylation of Pol2 (Pol2 0), and triptolide (TRP; 1 μM, 3 h) leads to degradation of Pol2. Both DRB and TRP decrease the amount of high molecular mass SUMO2/3-modified proteins (anti-SUMO2/3). **f** DRB and TRP reduce the average promoter SUMO2/3 ChIP-seq signal

To analyze both SUMO2/3 and PIAS1 chromatin binding in the context of transcription, we mapped chromatin occupancy of Pol2 in control, HS and recovery conditions in VCaP cells using ChIP-seq. HS markedly increased Pol2 signal at promoters and at 3’-untranslated regions of HS-induced genes, such as those in the HSP gene cluster (Fig. 6a), indicating a robust transcriptional activation of these genes. HS also resulted in accumulation of SUMO2/3 and PIAS1 onto the HSP gene promoters (Fig. 6a). After a recovery at normal growth temperature, the promoter PIAS1 disappeared, whereas the promoter SUMO2/3 and Pol2 signals persisted. Heat map analysis of the ChIP-seq signals at all TSSs revealed a positive correlation between SUMO2/3 and Pol2 in control, HS and recovery conditions, but for PIAS1 and Pol2 only in HS (Fig. 6b). Among Pol2-bound genes (promoter Pol2 RPKM >0.2), the promoter SUMO2/3 signal increased from control to HS to recovery (Fig. 6c). In contrast, among genes without Pol2 (promoter Pol2 RPKM ≤0.2), SUMO2/3 and PIAS1 signals were below the background level and did not change during HS or recovery. We next ranked Pol2-bound genes to four groups of increasing promoter Pol2 signal and analyzed the promoter signals of SUMO2/3 and PIAS1 within each group. Promoter SUMO2/3 signal increased with Pol2 promoter occupancy in control (r_s_ = 0.49, *P* <0.0001), HS (r_s_ = 0.51, *P* <0.0001), and recovery (r_s_ = 0.52, *P* <0.0001) conditions (Fig. 6c). A corresponding analysis for promoter PIAS1 signal revealed a robust positive correlation with Pol2 only in HS (Fig. 6d; r_s_ = 52, *P* <0.0001). These data show that promoter Pol2 and SUMO2/3 correlate in all assayed conditions, whereas the recruitment of PIAS1 onto Pol2-bound promoters is a transient response to acute heat stress.

### Promoter SUMOylation is dependent on active transcription

Next, we asked if the promoter SUMO2/3 signal is dependent on active transcription and whether it is a common feature of Pol2-paused promoters. To this end, we analyzed chromatin SUMO2/3 using ChIP-seq in cells treated with either DRB (5,6-dichlorobenzimidazole 1-β-D-ribofuranoside) that induces Pol2 promoter-proximal pausing or triptolide (TRP) that targets Pol2 to degradation [30]. DRB treatment efficiently removed hyperphosphorylated Pol2 (Fig. 6e; anti-Pol2: Pol2 0) from VCaP cells, while TRP treatment led to degradation of both hyperphosphorylated and non-phosphorylated Pol2 (anti-Pol2: Pol2 0 and Pol2 A). Interestingly, both DRB and TRP also decreased the amount of high molecular mass SUMOylated proteins (Fig. 6e; anti-SUMO2/3), further emphasizing that active transcription is needed for accumulation of SUMOylated proteins. Notably, DRB or TRP diminished promoter SUMO2/3 signal (Fig. 6f). These results suggest that induction of Pol2 promoter-proximal pausing with DRB does not increase promoter SUMO2/3 signal. Furthermore, promoter SUMO2/3 signal is dependent on active transcription, as inhibition of transcription with TRP leads to a decrease in SUMO2/3 at promoters.

### SUMOylation constrains HS-induced gene expression

**Fig. 7:**
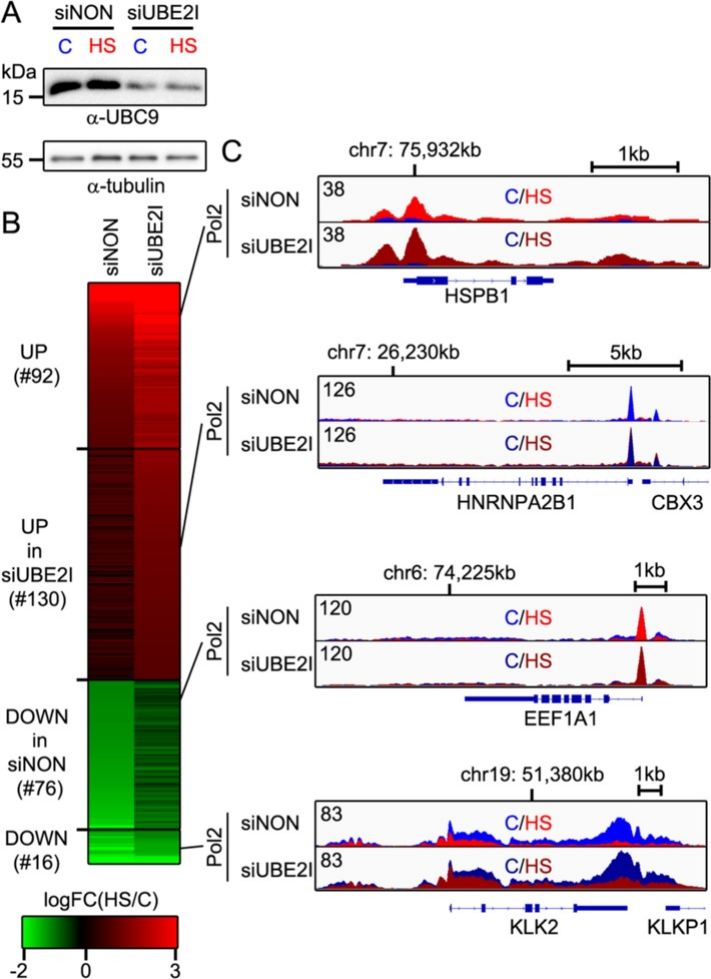
SUMOylation restricts HS-induced gene expression. **a** Immunoblot analysis of UBC9 levels from non-targeting siRNA (siNON)- and siUBE2I (gene for UBC9)-transfected VCaP cells. **b** Heat map showing HS-induced gene expression changes measured form Pol2 ChIP-seq in siNON- and siUBE2I-transfected cells (log_2_ fold change [HS/C]). Ninety-two genes were induced (shades of red) in both samples (UP; FDR <0.01, log_2_ fold change >1) and 130 genes exclusively in siUBE2I (UP in siUBE2I). Seventy-six genes were repressed (shades of green) exclusively in siNON (DOWN in siNON; FDR <0.01, log_2_ fold change <-1), and 16 genes in both siNON and siUBE2I (DOWN). **c** Examples of Pol2 signal along genes in different categories in (b): *HSPB1* (category: UP), *HNRNPA2B1* (UP in siUBE2I), *EEF1A1* (DOWN in siNON), and *KLK2* (DOWN). Pol2 ChIP-seq signal control (C; blue) and HS (red) conditions from siNON (lighter colors) and siUBE2I (darker colors). Numbers indicate maximal signal

To reveal the functional effect of SUMOylation on the regulation of HS-induced genes, we silenced *UBE2I* (gene for UBC9) in VCaP cells with siRNA (siUBE2I) and used ChIP-seq to map the genome-wide binding of Pol2 in control (C) and HS conditions. A marked reduction of UBC9 protein level was seen in siUBE2I compared to siNON (Fig. 7a). HS-induced changes in transcription were analyzed from Pol2 ChIP-seq signal. Using stringent criteria (FDR <0.01, fold change >2), HS induced 92 genes (Fig. 7b UP; for example, *HSPB1* in Fig. 7c) and repressed 16 genes (Fig. 7b DOWN; for example, *KLK2* in Fig. 7c) in siNON- and siUBE2I-transfected cells. As expected, HS-induced genes were enriched in GO term categories of unfolded proteins and stress (Additional file 4: Table S3). In addition, 130 genes enriched in GO terms of protein folding and cellular metabolic processes were HS-induced exclusively in siUBE2I-transfected cells (Fig. 7b UP in siUBE2I; for example, *HNRNPA2B1* Fig. 7c), and 76 genes enriched in GO terms of translation and metabolic processes were HS-repressed exclusively in siNON-transfected cells (Fig. 7b DOWN in siNON; for example, *EEF1A1* Fig. 7c). These data suggest that impaired SUMOylation leads to a more pronounced transcription activation of HS-induced genes and attenuation of HS-repression.

**Fig. 8:**
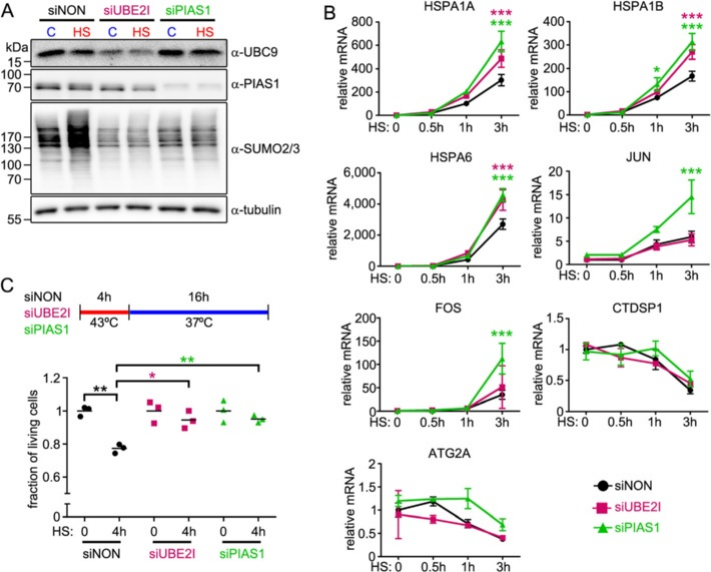
**a** Anti-UBC9, anti-PIAS1, and anti-SUMO2/3 antibody immunoblotting of total cellular lysates from control (siNON), *UBE2I*- (siUBE2I), or *PIAS1*-silenced (siPIAS1) VCaP cells in C (37 °C) or HS (30 min at 43 °C) conditions. **b** Effect of UBC9 or PIAS1 depletion on the gene expression of select HS-inducible (*HSPA1A*, *HSPA1B*, *HSPA6*, *JUN*, *FOS*) and HS-repressed (*CTDSP1*, *ATG2A*) target genes. Quantitation of mRNA was done using RT-qPCR with target- specific primers. Measurements were normalized to *GAPDH* mRNA levels, and fold changes were calculated in reference to siNON at control conditions. Error bars are standard deviations of three independent biological replicates. Stars depict statistical significances of pair-wise comparisons of each time point (purple, siUBE2I vs. siNON; green, siPIAS1 vs. siNON; *P <0.01, ***P <0.001; ANOVA and Bonferroni). **c** Cell survival after HS. siUBE2I-, siPIAS1-, and siNON-transfected cells were exposed to HS (43 °C) for 4 h and allowed to recover at 37 °C for 16 h, and the proportions of living cells in the samples were measured and normalized to non-HS (HS: 0). Three biological replicates are shown (black = siNON, purple = siUBE2I, green = siPIAS1) and the bars represent the mean values of each group. Stars depict statistical significances of indicated pair-wise comparisons (*P <0.05, **P <0.01; ANOVA and Bonferroni)

Next, we used RT-qPCR to more specifically examine the effect of SUMOylation during a HS time course. As shown in Fig. 8a, HS-induced cellular SUMOylation was abolished in siUBE2I-transfected cells, and it was markedly diminished in siPIAS1-transfected cells (Fig. 8a; α-SUMO-2/3). Cellular RNA was isolated from siNON-, siUBE2I-, or siPIAS1-transfected cells after different periods of HS exposure (0, 0.5, 1, 3 h) and the expression of putative HS-inducible genes *HSPA1A*, *HSPA1B*, *HSPA6*, *JUN*, and *FOS* was measured using RT-qPCR. All candidate HS-inducible genes showed a HS time-dependent increase in expression. Interestingly, silencing of either UBC9 or PIAS1 resulted in even more robust expression of *HSPA1A*, *HSPA1B*, and *HSPA6*, whereas only silencing of PIAS1 augmented the expression of *JUN* and *FOS* (Fig. 8b). Expression of genes that were HS-repressed *CTDSP1* and *ATG2A* did not change in siUBE2I- or siPIAS1-transfected cells (Fig. 8b). These data imply that HS-induced genes are repressed by a promoter SUMOylation-linked mechanism that involves activation of PIAS1.

Since attenuation of SUMOylation machinery resulted in augmented induction of HSP genes, we next analyzed whether this influences survival from HS. VCaP cells were first exposed to HS for 4 h and subsequently allowed to recover for 16 h at 37 °C before measuring the proportion of living cells [31]. Interestingly, the silencing of UBC9 or PIAS1 had a small, but statistically significant decreasing effect on the number of dead cells (Fig. 8c), indicating that silencing of the SUMOylation machinery provides a survival advantage for cells exposed to heat stress, probably due to enhanced HSP induction.

## Discussion

According to recent proteome-wide studies, a large number of proteins that are involved in nuclear processes are SUMOylated as a swift response to proteotoxic cell stress [8–10]. However, the biological impact of this SUMO response has remained elusive, albeit it has been suggested to support cell viability [8]. Here, we demonstrate by using genome-wide approaches that HS induces a rapid reorganization of chromatin-bound SUMO2/3 in a fashion that positively correlates with transcriptional activity at promoters and enhancers. SUMO2/3 accumulated in HS-exposed K562 cells at genomic regions, especially promoters, that under normal cell growth conditions, represent active, open chromatin. SUMO2/3 signal was restricted to relatively narrow chromatin regions likely reflecting occupancy of SUMOylated TFs on the chromatin. Multiple TFs are known to co-bind at enhancers in a cooperative manner that is thought to integrate transcriptional signals [18, 32]. Active enhancers are transcribed [27], and although the importance of this eRNA transcription is still under debate, the amount of transcription is associated with enhancer activity [26]. The extensive overlap between TF binding (ENCODE data) and SUMO2/3 in non-stressed K562 cells may indicate the role of SUMOylation in the regulation of protein-protein interactions at TF complexes. HS induced an increase in SUMOylation and eRNA transcription at intergenic SUMO2/3 binding sites, suggesting that these sites are HS-induced enhancers. Furthermore, in HS, the genes associated with intergenic SUMO2/3 sites were also more actively transcribed than unassociated genes, further linking the intergenic chromatin SUMOylation to the regulation of enhancer activity in acute heat stress. The major HS-activated TF, HSF1, co-occurred with SUMO2/3 on chromatin in heat-shocked cells, suggesting that HSF1 is one of the SUMO2/3-regulated TFs on chromatin. In contrast, a loss of SUMO2/3 from chromatin in response to acute heat stress occurred at non-transcribed loci without active histone marks, but with co-occurrence of histone methyltransferase and CTCF-cohesin complexes. Since proteins in both complexes are differentially SUMOylated in cell stress [9, 24, 33], the SUMOylation status may regulate their complex formation and have an impact on chromatin methylation and architecture. These changes could be linked to HS-induced rearrangements in chromatin architecture that have been recently reported in *D melanogaster* [34].

Pol2 and Pol3 promoters have been shown to undergo SUMOylation in a gene activity-correlating manner in proliferating fibroblasts [14]. Our genome-wide analyses confirm that SUMO2/3 is widely present at promoters of actively transcribed genes under non-stress conditions and that Pol3-binding sites have a nearly complete overlap with SUMO2/3. Upon stress, the amount of promoter SUMO2/3 substantially increases at Pol2 promoters, while it is attenuated at Pol3 binding sites, suggesting that the Pol2 and Pol3 machineries are differentially regulated by stress-inducible SUMOylation. In line with previous results [1, 2], acute heat stress induced rapid activation of HSP genes, despite a general repression of transcription. A simultaneous increase in Pol2 pausing implies that it is a major mechanism for transcriptional repression in HS, as has been shown before [28, 29]. Both transcription repression and Pol2 pausing were stronger in promoter SUMO2/3-associated genes than genes without promoter SUMO2/3. Because several proteins that regulate Pol2 pausing, for example, CCNT1 of positive transcription elongation factor b (P-TEFb) and NELF-A and NELF-E of negative elongation complex [9], have been identified as SUMO2/3 targets whose modification level increases in HS, it is an intriguing possibility that the global stress-caused Pol2 pausing is controlled by SUMOylation of these regulatory proteins. The difference between HS-induced Pol2- and Pol3-associated SUMOylation could also be explained by increased SUMOylation of Pol2-specific regulatory proteins, such as NELF or P-TEFb.

Chromatin SUMOylation has been reported to govern coordinated repression of histone and growth regulatory genes [14]. In senescent cells, chromatin SUMOylation is widely attenuated, with the SUMOylation machinery being largely lost from chromatin except for histone and tRNA clusters [14]. Of note, HS did not markedly influence chromatin SUMOylation of the regulatory regions in the latter two housekeeping gene clusters. These results suggest that, in contrast to a short term HS that exerts a transient and reversible repression of transcription, a permanent repression of gene programs, such as cell senescence, leads to a loss of promoter SUMOylation. In comparison to HS-induced alterations in gene activity, the gene re-programming in senescence occurs in a slower pace and is likely to involve more dramatic alterations in TF activity, histone marks, and chromatin state.

Insightful studies in yeast have also associated recruitment of SUMO to promoters of activated genes [35] and suggested that the role of promoter SUMOylation is to inhibit transcription re-initiation of inducible genes [36]. Our finding that a partial inactivation of SUMOylation machinery by depleting UBC9 or PIAS1 resulted in a more pronounced activation of HSP genes in HS supports this model. However, since increased promoter SUMOylation occurs also at promoters of the repressed genes, the increased promoter SUMOylation is not simply a result of augmented gene transcription. Nevertheless, a common feature of both up- and downregulated genes in HS, is the increased promoter-proximal transcription. We suggest that active transcription initiation or elongation licensing [37], not necessarily continued re-initiation, as likely candidates for SUMOylation-regulated processes. These notions are also supported by the loss of promoter SUMOylation when transcription was inhibited using Pol2 inhibitors (DRB or TRP).

HS-triggered SUMOylation has been suggested to arise from activation of SUMO ligases and deactivation of SUMO proteases [38]. HS induces marked chromatin binding of the SUMO ligase PIAS1. This is intriguing, as PIAS1 has not been previously connected to cellular stress responses. Our data suggest that PIAS1 functions as one of the HS-activated SUMO ligases and that active SUMOylation occurs on chromatin in HS. However, as not all HS-acquired SUMO2/3 sites co-occurred with PIAS1, proteins are also likely to be SUMOylated in a PIAS1-independent manner or recruited onto chromatin in a pre-SUMOylated state. Since the occupancy of PIAS1 on chromatin after HS was more transient than that of SUMO2/3, activation and recruitment of SUMO proteases onto chromatin [39], is perhaps needed for reversal of chromatin SUMOylation when cell stress is alleviated.

## Conclusions

We show that acute stress-induced SUMOylation of chromatin-bound proteins occurs at actively transcribed promoters and enhancers. SUMOylation in heat shock correlates with Pol2 pausing and represses transcriptional activity of HSP genes. Our genome-wide data also reveal that the global promoter SUMOylation is not only restricted to the genes that are either stimulated or repressed by acute heat stress, but is a more general phenomenon linked to transcription regulation at promoters and enhancers. Upon stress, the amount of promoter Pol2 and SUMO2/3 increases if the gene is activated or if the Pol2 is paused at the promoter. Stress-induced chromatin SUMOylation is initiated by HSF1-independent activation and chromatin binding of SUMOylation machinery, which targets transcription regulatory proteins. At promoters, SUMO2/3 recruitment is dependent on transcriptional activity, but its function is to restrict transcription. Promoter SUMOylation is likely to regulate transcription by targeting Pol2 regulatory proteins in HS.

## Methods

### Cells culture and treatments

K562 cells (European Collection of Cell Cultures) were grown in RPMI1640 (Gibco) supplemented with 10 % fetal bovine serum, 1 % penicillin/streptomycin, and 2 mM l-glutamine. VCaP cells (American Type Culture Collection) were grown as previously described [15]. For ChIP-seq cells were heat-shocked in 43 °C incubator and for GRO-seq in 43 °C water bath for indicated times. In recovery experiments, VCaP cells were first heat shocked for 30 min at 43 °C and then returned to 37 °C for 60 min. Pol2 inhibitors DRB (5,6-dichlorobenzimidazole 1-β-D-ribofuranoside; Sigma-Aldrich; 100 μM) and triptolide (Sigma-Aldrich; 1 μM) were applied to cells 3 h prior to continuing with ChIP-seq protocol. In viability assay, siRNA treated cells were exposed to 4 h HS at 43 °C. After 16 h recovery at 37 °C, cells were collected, stained with propidium iodide (Biotool, USA), and analyzed using flow cytometer (FACSCalibur, BD biosciences). Proportion of live cells was further normalized to non HS control in each siRNA, and differences between groups of three biological replicates were analyzed in GraphPad Prism (one-way ANOVA and Bonferroni’s multiple testing correction).

### Antibodies

Antibodies used for immunoblotting were anti-SUMO2/3 (M114-3, MBL), anti-UBC9 (sc10759, Santa Cruz Biotechnology), anti-PIAS1 (ab77231, Abcam), anti-tubulin (sc5286, Santa Cruz Biotechnology), anti-laminB1 (sc6216, Santa Cruz Biotechnology), anti-Pol2 (sc-899, Santa Cruz Biotechnology), anti-histone H3 (ab1791, Abcam), and anti-HSF1 (ADI-SPA-901; ENZO Life Sciences). Antibodies used for ChIP-seq were anti-SUMO2/3 (M114-3, MBL), anti-PIAS1 (ab77231, Abcam), and anti-Pol2 (MMS-126R, Covance).

### ChIP-seq libraries

ChIP-seq was done with minor modifications from a published protocol [13]. Briefly, the cells grown on 10 cm dishes were fixed with formaldehyde at room temperature (final concentration 1 % v/v; 8 min for K562 cells and 10 min for VCaP cells), and cross-linking was stopped with glycine (final concentration 125 mM). Chromatin was fragmented to approximately 200 to 500 bp using sonication (Bioruptor UCD-300, Diagenode). Target antibodies (Ab) were coupled to protein-A or -G beads (Millipore), fragmented chromatin was incubated with Ab-coupled beads overnight, washed, eluted, and de-cross-linked in the presence of proteinase K (Fermentas). Chromatin fragments were purified using MinElute columns (Qiagen), ChIP-seq libraries were prepared using NEBNext kit (New England Biolabs) and sequenced at BGI (Hong Kong, China; K562 SUMO2/3 control and HS; VCaP Pol2 control) or at EMBL genomics core facility (Heidelberg, Germany; all other samples). Two biological replicates were made for each sample. Fragmented de-cross-linked chromatin was used as a control for K562 samples and previously published IgG ChIP-seq chromatin for VCaP samples [15].

### GRO-seq libraries

Samples were produced as two biological replicates essentially as described before [40]. Briefly, nuclei from approximately 10 million cells were collected in swelling buffer, run-on reactions were done in the presence of Br-UTP, RNA was isolated using TRIZOL-LS reagent (Life Technologies), and labeled RNA molecules were affinity purified using agarose-conjugated anti-BrdU (sc32323AC, Santa Cruz Biotechnology). Labeled RNAs were processed for next-generation sequencing with minor modifications from a published protocol [41]. Samples were sequenced with HiSeq 2000 at EMBL genomics core facility (Heidelberg, Germany).

### RNA interference and RT-qPCR

HSF1 was silenced from K562 cells as described before [19]. Briefly, cells were transfected using electroporation with plasmids expressing short hairpin-RNA against HSF1 (shHSF1) or scrambled sequence (shSCR). VCaP cells were reverse-transfected with siRNAs against *UBE2I* (gene for UBC9; siUBE2I), PIAS1 (siPIAS1), or non-targeting control (siNON) siRNA (Dharmacon; On-TARGETplus pools and non-targeting pool) on 6-well plates using Lipofectamine RNAiMAX transfection reagent (Life Technologies). Four days after transfection, VCaP cells were heat shocked (0 min, 30 min, 1 h, 3 h), total cellular RNA was collected (TriPure reagent; Roche), and cDNA was synthesized with oligo dT primers (Transcriptor First Strand cDNA synthesis Kit; Roche). Three biological replicates were analyzed using LightCycler 460 SYBR GREEN I reagent and LightCycler 480 PCR apparatus (Roche). GAPDH was used to normalize amounts of mRNA between samples, and non-stressed siNON sample to get the relative mRNA levels in each sample. Primer sequences are available upon request. Statistical significance of the changes in RT-qPCR data were analyzed using one-way ANOVA and Bonferroni’s multiple testing correction in GraphPad Prism.

### Data analysis

#### ChIP-seq

Sequenced raw reads were quality controlled using FastQC [42] and quality filtered using FASTX-toolkit [43] before reads were mapped to human genome (hg19) using bowtie [44] keeping only uniquely mapping reads. Initial binding sites (peaks) were defined for both biological replicates against control sample using findPeaks program of Homer package [45] with default settings. In addition, in K562 cells, peaks with small number of reads in SUMO2/3 ChIP-seq samples were removed (control: <9 reads, HS: <10 reads). Only areas where peak was defined in both biological replicates were considered representative for the given condition and used in analysis. SUMO2/3 peaks were associated with nearest genes using HOMER. All ChIP-seq datasets were normalized to 10^7^ reads for visualization and ChIP-seq signal comparison analysis. Signal matrixes for heat maps and line profiles were done in HOMER and visualized using imageJ [46] and R [47]. Chromatin tracks were done in IGV [48] and assembled in Photoshop (Adobe). DNA motif discovery was done with findMotifsGenome program of the HOMER package. SUMO2/3 peaks with four-fold more tags in HS (HS-enriched) or in control (HS-depleted) were used in GO-term enrichment analysis. Gene Ontology (GO) enrichment analysis was done in GREAT v. 2.0.2 [49] using default region settings. Venn diagrams were produced using Venn Diagram Plotter [50] and VENNY [51]. Analysis of transcription from siNON and siUBE2I Pol2 ChIP-seq was done by counting Pol2 tags from TSS to TTS +3 kb area using HOMER and by analyzing differential expression using edgeR. Criteria for differential expression in HS versus control samples were FDR <0.01 and fold change >2. Gene Ontology (GO) term enrichment analysis of HS-induced and HS-repressed gene groups determined using Pol2 ChIP-seq in VCaP cells was done in DAVID [52].

#### GRO-seq

GRO-seq reads were quality controlled using FastQC and FASTX-toolkit (minimum 97 % of bps over quality score 10). Poly-A tails were trimmed, and smaller than 25 nt long reads and reads that mapped to rRNA were discarded. Remaining reads were mapped to reference human genome (hg19) using bowtie (v = 2, m = 3, k = 1). Promoter (TSS to +250 bp) and gene body (+250 bp to TTS) transcription in was analyzed using HOMER. Gene body RPKM cutoff >0.5 was used to differentiate between transcribed and non-transcribed genes. Promoter-proximal pausing index (PPI) was calculated as ratio of RPKMs at promoter and gene body GRO-seq transcription. Differential expression (FDR <0.01 and fold change >2 as cutoffs) for transcribed genes was analyzed using edgeR [53]. Gene Onthology (GO) term enrichment for HS-induced and -repressed genes was done using DAVID [52] and GOTERM_BP_FAT annotations. In order to determine transcribed enhancers, K562 GRO-seq libraries were combined and intergenic transcripts (over 10 kb from the TTS to avoid gene read through artifacts) were determined using HOMER (findPeaks with GRO-seq *de novo* transcript detection). Chromatin loci where two opposing strand transcripts with no more than two-fold difference in read count (±500 bp from the center) were identified no more than 1 kb apart from each other, were considered eRNA enhancers. Transcription and SUMO2/3 signal was measured from 1 kb window centered to eRNA enhancers. Kruskal-Wallis statistical test and Dunn’s post test were done in GraphPad Prism software to evaluate statistical significance of the changes in SUMO2/3 signals in different transcription categories (gene body transcription and eRNA enhancer transcription).

#### External datasets

K562 cell specific hg19 mapped ChIP-seq and DNaseI data released in the ENCODE project were used in the analysis (ENCODE project consortium, 2011; full list of used datasets in Additional file 5: Table S4). In heat maps and line profiles, the signal from replicates was combined and normalized to 10^7^ mapped reads. In SUMO2/3 co-occurrence analysis, K562 specific ChIP-seq datasets from transcription factor super-track (release 3 August 2013) were used [18]. VCaP SUMO2/3 and PIAS1 ChIP-seq data from control conditions (GEO accession: GSE56086) [15] were analyzed in the same way as other VCaP ChIP-seq data described above. Peak detection for HSF1 and HSF2 (GEO accession: GSE43579) [19] was done with HOMER using IgG ChIP-seq in HS as a control. Pol3-binding sites were determined with HOMER from hg19 mapped POLR3G ChIP-seq data (the ENCODE project, Snyder-lab) using appropriate control.

### Data

ChIP-seq and GRO-seq datasets are available with accession code: GSE66448 in GEO database.

## Additional files

Additional file 1:
**Supplementary Figs. S1–S6.** (PDF 335 kb) Additional file 2: Table S1. SUMO2/3 enriched motifs in control and HS treated K562 cells. (XLSX 44 kb) Additional file 3: Table S2. Co-occurrence of SUMO2/3 peaks and ENCODE determined transcription factor binding in K562 cells. (XLSX 18 kb) Additional file 4: Table S3. Gene ontology (GO) - term enrichment in GRO-seq HS-induced and HS-repressed genes, and HS-enriched and HS-depleted SUMO2/3 peaks in K562 cells. GO-term enrichment of Pol2 HS-induced and HS-repressed genes in VCaP cells. (XLSX 129 kb) Additional file 5: Table S4. Details of external datasets used in the analyses. (XLSX 13 kb)

## Abbreviations

ChIP-seq: chromatin immunoprecipitation-coupled to deep sequencing
DRP: 5,6-dichlorobenzimidazole 1-β-D-ribofuranoside
GRO-seq: global run-on sequencing
HS: heat shock
HSF: heat shock factor
TF: transcription factor
TRP: triptolide
PPI: promoter-proximal pausing index
RPKM: reads per kilobase per million mapped reads

## References

[1] Lindquist S (1986). The heat-shock response. Annu Rev Biochem.

[2] Sonna LA, Fujita J, Gaffin SL, Lilly CM. Invited review: effects of heat and cold stress on mammalian gene expression. J Appl Physiol (1985). 2002;92:1725–42. 10.1152/japplphysiol.01143.2001 11896043

[3] Morimoto RI (1998). Regulation of the heat shock transcriptional response: cross talk between a family of heat shock factors, molecular chaperones, and negative regulators. Genes Dev.

[4] Åkerfelt M, Morimoto RI, Sistonen L (2010). Heat shock factors: integrators of cell stress, development and lifespan. Nat Rev Mol Cell Biol.

[5] Flotho A, Melchior F (2013). Sumoylation: a regulatory protein modification in health and disease. Annu Rev Biochem.

[6] Zhang FP, Mikkonen L, Toppari J, Palvimo JJ, Thesleff I, Jänne OA (2008). Sumo-1 function is dispensable in normal mouse development. Mol Cell Biol.

[7] Wang L, Wansleeben C, Zhao S, Miao P, Paschen W, Yang W (2014). SUMO2 is essential while SUMO3 is dispensable for mouse embryonic development. EMBO Rep.

[8] Golebiowski F, Matic I, Tatham MH, Cole C, Yin Y, Nakamura A, et al. System-wide changes to SUMO modifications in response to heat shock. Sci Signal. 2009;2:ra24. 10.1126/scisignal.2000282 19471022

[9] Hendriks IA, D'Souza RC, Yang B, Verlaan-de Vries M, Mann M, Vertegaal AC (2014). Uncovering global SUMOylation signaling networks in a site-specific manner. Nat Struct Mol Biol.

[10] Tammsalu T, Matic I, Jaffray EG, Ibrahim AF, Tatham MH, Hay RT. Proteome-wide identification of SUMO2 modification sites. Sci Signal. 2014;7:rs2. 10.1126/scisignal.2005146 PMC4051997 24782567

[11] Anckar J, Sistonen L (2007). Heat shock factor 1 as a coordinator of stress and developmental pathways. Adv Exp Med Biol.

[12] Rytinki M, Kaikkonen S, Sutinen P, Paakinaho V, Rahkama V, Palvimo JJ (2012). Dynamic SUMOylation is linked to the activity cycles of androgen receptor in the cell nucleus. Mol Cell Biol.

[13] Paakinaho V, Kaikkonen S, Makkonen H, Benes V, Palvimo JJ (2014). SUMOylation regulates the chromatin occupancy and anti-proliferative gene programs of glucocorticoid receptor. Nucleic Acids Res.

[14] Neyret-Kahn H, Benhamed M, Ye T, Le Gras S, Cossec JC, Lapaquette P (2013). Sumoylation at chromatin governs coordinated repression of a transcriptional program essential for cell growth and proliferation. Genome Res.

[15] Toropainen S, Malinen M, Kaikkonen S, Rytinki M, Jääskeläinen T, Sahu B (2015). SUMO ligase PIAS1 functions as a target gene selective androgen receptor coregulator on prostate cancer cell chromatin. Nucleic Acids Res.

[16] ENCODE Project Consortium (2012). An integrated encyclopedia of DNA elements in the human genome. Nature.

[17] Zhou VW, Goren A, Bernstein BE (2011). Charting histone modifications and the functional organization of mammalian genomes. Nat Rev Genet.

[18] Gerstein MB, Kundaje A, Hariharan M, Landt SG, Yan KK, Cheng C (2012). Architecture of the human regulatory network derived from ENCODE data. Nature.

[19] Vihervaara A, Sergelius C, Vasara J, Blom MA, Elsing AN, Roos-Mattjus P (2013). Transcriptional response to stress in the dynamic chromatin environment of cycling and mitotic cells. Proc Natl Acad Sci U S A.

[20] Ong CT, Corces VG (2014). CTCF: an architectural protein bridging genome topology and function. Nat Rev Genet.

[21] Xie D, Boyle AP, Wu L, Zhai J, Kawli T, Snyder M (2013). Dynamic trans-acting factor colocalization in human cells. Cell.

[22] Frietze S, O'Geen H, Blahnik KR, Jin VX, Farnham PJ (2010). ZNF274 recruits the histone methyltransferase SETDB1 to the 3' ends of ZNF genes. PLoS One.

[23] Heidari N, Phanstiel DH, He C, Grubert F, Jahanbani F, Kasowski M (2014). Genome-wide map of regulatory interactions in the human genome. Genome Res.

[24] Ivanov AV, Peng H, Yurchenko V, Yap KL, Negorev DG, Schultz DC (2007). PHD domain-mediated E3 ligase activity directs intramolecular sumoylation of an adjacent bromodomain required for gene silencing. Mol Cell.

[25] Core LJ, Waterfall JJ, Lis JT (2008). Nascent RNA sequencing reveals widespread pausing and divergent initiation at human promoters. Science.

[26] Kim TK, Hemberg M, Gray JM, Costa AM, Bear DM, Wu J (2010). Widespread transcription at neuronal activity-regulated enhancers. Nature.

[27] De Santa F, Barozzi I, Mietton F, Ghisletti S, Polletti S, Tusi BK (2010). A large fraction of extragenic RNA pol II transcription sites overlap enhancers. PLoS Biol.

[28] Gilmour DS, Lis JT (1986). RNA polymerase II interacts with the promoter region of the noninduced hsp70 gene in Drosophila melanogaster cells. Mol Cell Biol.

[29] Adelman K, Lis JT (2012). Promoter-proximal pausing of RNA polymerase II: emerging roles in metazoans. Nat Rev Genet.

[30] Bensaude O (2011). Inhibiting eukaryotic transcription: Which compound to choose? How to evaluate its activity?. Transcription.

[31] Elsing AN, Aspelin C, Björk JK, Bergman HA, Himanen SV, Kallio MJ (2014). Expression of HSF2 decreases in mitosis to enable stress-inducible transcription and cell survival. J Cell Biol.

[32] Siersbaek R, Rabiee A, Nielsen R, Sidoli S, Traynor S, Loft A (2014). Transcription factor cooperativity in early adipogenic hotspots and super-enhancers. Cell Rep.

[33] Wang J, Wang Y, Lu L (2012). De-SUMOylation of CCCTC binding factor (CTCF) in hypoxic stress-induced human corneal epithelial cells. J Biol Chem.

[34] Li L, Lyu X, Hou C, Takenaka N, Nguyen HQ, Ong CT (2015). Widespread rearrangement of 3D chromatin organization underlies polycomb-mediated stress-induced silencing. Mol Cell.

[35] Rosonina E, Duncan SM, Manley JL (2010). SUMO functions in constitutive transcription and during activation of inducible genes in yeast. Genes Dev.

[36] Rosonina E, Duncan SM, Manley JL (2012). Sumoylation of transcription factor Gcn4 facilitates its Srb10-mediated clearance from promoters in yeast. Genes Dev.

[37] Fuda NJ, Ardehali MB, Lis JT (2009). Defining mechanisms that regulate RNA polymerase II transcription in vivo. Nature.

[38] Lewicki MC, Srikumar T, Johnson E, Raught B (2014). The S. cerevisiae SUMO stress response is a conjugation-deconjugation cycle that targets the transcription machinery. J Proteomics.

[39] Nayak A, Muller S (2014). SUMO-specific proteases/isopeptidases: SENPs and beyond. Genome Biol.

[40] Kaikkonen MU, Niskanen H, Romanoski CE, Kansanen E, Kivelä AM, Laitalainen J (2014). Control of VEGF-A transcriptional programs by pausing and genomic compartmentalization. Nucleic Acids Res.

[41] Ingolia NT, Ghaemmaghami S, Newman JR, Weissman JS (2009). Genome-wide analysis in vivo of translation with nucleotide resolution using ribosome profiling. Science.

[42] Andrews, S. FastQC A quality control tool for high throughput sequence data. Available at: http://www.bioinformatics.babraham.ac.uk/projects/fastqc/.

[43] Hannon Lab: FASTX Toolkit. Available at: http://hannonlab.cshl.edu/fastx_toolkit/index.html.

[44] Langmead B, Trapnell C, Pop M, Salzberg SL (2009). Ultrafast and memory-efficient alignment of short DNA sequences to the human genome. Genome Biol.

[45] Heinz S, Benner C, Spann N, Bertolino E, Lin YC, Laslo P (2010). Simple combinations of lineage-determining transcription factors prime cis-regulatory elements required for macrophage and B cell identities. Mol Cell.

[46] Schneider CA, Rasband WS, Eliceiri KW (2012). NIH Image to ImageJ: 25 years of image analysis. Nat Methods.

[47] Development Core Team R (2012). R: A language and environment for statistical computing.

[48] Robinson JT, Thorvaldsdottir H, Winckler W, Guttman M, Lander ES, Getz G (2011). Integrative genomics viewer. Nat Biotechnol.

[49] McLean CY, Bristor D, Hiller M, Clarke SL, Schaar BT, Lowe CB (2010). GREAT improves functional interpretation of cis-regulatory regions. Nat Biotechnol.

[50] Littlefield K, Monroe M. Venn diagram plotter. Available at: http://omics.pnl.gov/software/venn-diagram-plotter.

[51] Oliveros JC. VENNY. An interactive tool for comparing lists with Venn diagrams. Available at: http://bioinfogp.cnb.csic.es/tools/venny/index.html.

[52] da Huang W, Sherman BT, Lempicki RA (2009). Systematic and integrative analysis of large gene lists using DAVID bioinformatics resources. Nat Protoc.

[53] Robinson MD, McCarthy DJ, Smyth GK (2010). edgeR: a Bioconductor package for differential expression analysis of digital gene expression data. Bioinformatics.

