# Supplementary material for: Global SUMOylation on active chromatin is an acute heat stress response restricting transcription
Source: Genome Biol. 2015 Jul 28;16(1):153. doi: 10.1186/s13059-015-0717-y (PMC4531811; doi:10.1186/s13059-015-0717-y)
Supplement: Additional file 1: — Supplementary Figs. S1–S6. (PDF 335 kb) [file 13059_2015_717_MOESM1_ESM.pdf]

Additional file 1, supplementary figures

Global SUMOylation on active chromatin is an acute heat stress response restricting transcription

Einari A. Niskanen, Marjo Malinen, Päivi Sutinen, Sari Toropainen, Ville Paakinaho, Anniina Vihervaara, Jenny Joutsen, Minna U. Kaikkonen, Lea Sistonen, Jorma J. Palvimo

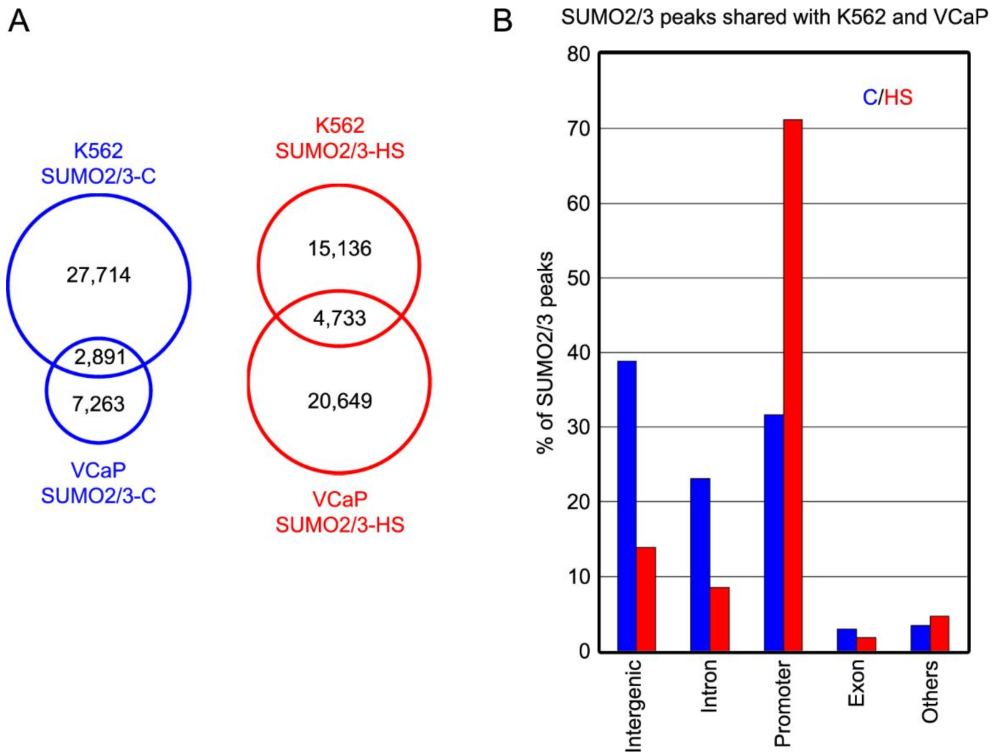

Figure S1 Comparison of SUMO2/3 peaks in K562 and VCaP cells. (A) Venn diagrams showing overlap of K562 and VCaP SUMO2/3 peaks in control (C) and HS conditions. (B) Distribution of SUMO2/3 peaks shared between K562 and VCaP in control (blue) or HS (red) conditions in annotated genomic loci.

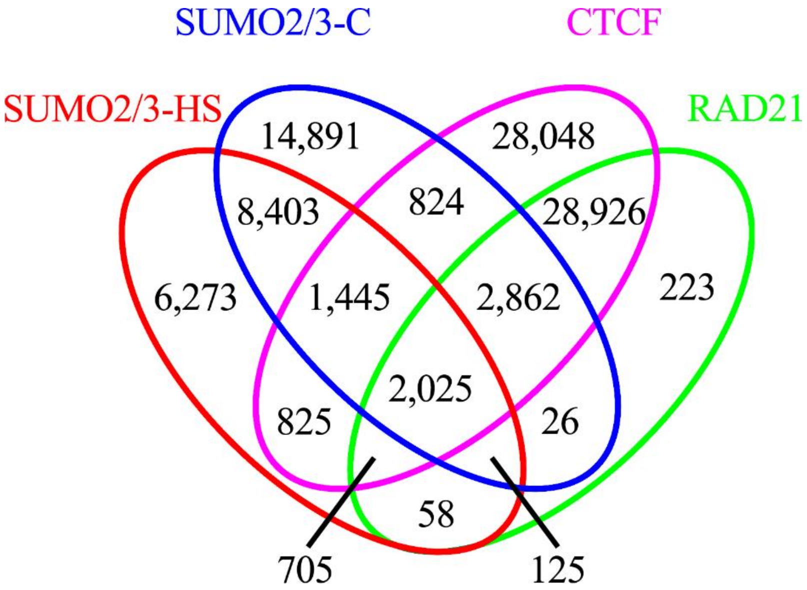

Figure S2 Venn diagram showing co-occurrence of K562 cell SUMO2/2 control condition unique peaks with CTCF, Rad21, and ZNF143 peaks.

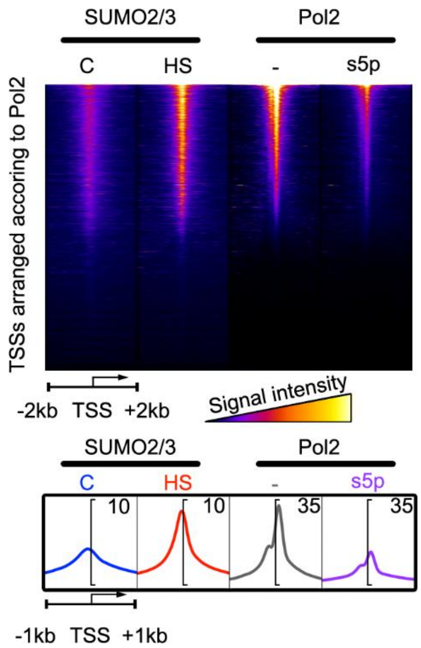

Figure S3 Promoter SUMO2/3 ChIP-seq signal correlates with Pol2 and serine 5-phosphorylated Pol2 (s5p). Heat map showing ChIP-seq signal intensities of K562 SUMO2/3 in control (C) and HS conditions, and Pol2 and Pol2-s5p at ±2kb window centered at transcription start sites (TSSs). TSSs are arranged according to Pol2 signal. Intensity is shown as a false-color scale (intensity increases from darker to brighter colors). Line profile of the average signal from the same ChIP-seq data sets at ±1kb region surrounding TSSs. Intensity of SUMO2/3 signal peaks in the "valley" between Pol2 local maxima.

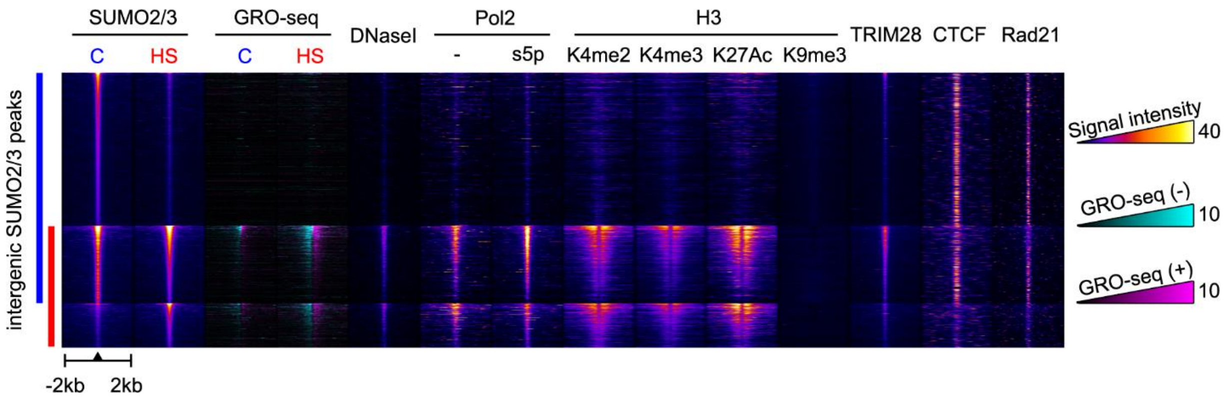

Figure S4 Chromatin environment surrounding intergenic K562 SUMO2/3 peaks. Heat map showing ChIP-seq and GRO-seq signal intensities at ±2kb window centered at intergenic K562 SUMO2/3 peaks in control (blue) and HS (red) conditions. K562 cell-specific DNaseI, Pol2, Pol2-s5p, histone, TRIM28, CTCF, and Rad21 data are from the ENCODE project (www.encodeproject.org). Intensities are depicted as a false-color scale (intensity increases from darker to brighter colors) for DNaseI and ChIP-seq data. GRO-seq signal is shown separately for negative (cyan) and positive (magenta) direction reads.

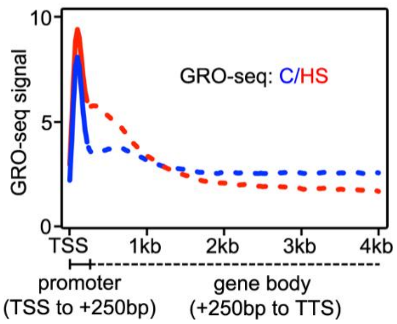

Figure S5 Average GRO-seq signal along the annotated genes in K562 cells. Average GRO-seq signal from positive-strand reads in control (blue) or HS (red) is shown at the first 4-kb region. GRO-seq signal peaked at the first 250 bp from the TSS and this region was used as the promoter proximal region when determining Pol2 promoter-pausing pausing index (PPI). The rest of the gene was used as the gene body signal in analysis of transcription.

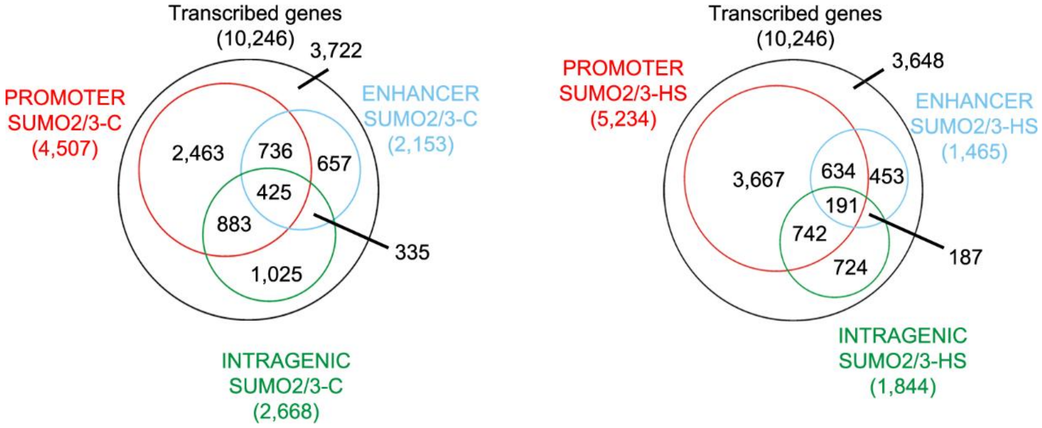

Figure S6 Association of SUMO2/3 peaks with transcriptionally active genes in K562 cells. Transcribed genes were divided according to their association to SUMO2/3 peaks: promoter SUMO2/3-associated (SUMO2/3 peak within -1kb to +100 from the annotated TSS), enhancer SUMO2/3-associated (intergenic SUMO2/3 peak within 100kb of the TSS), and intragenic SUMO2/-associated (intragenic SUMO2/3 peak). Venn diagrams indicate the number of genes in each SUMO2/3 association category in control (SUMO2/3-C) or HS (SUMO2/3-HS) conditions. Transcribed genes had gene body GRO-seq RPKM >0.5 in control or HS conditions.
